# Cumulative disadvantage? Exploring relationships between neighbourhood deprivation trends (1991 to 2006) and mortality in New Zealand

**DOI:** 10.1186/1476-072X-12-38

**Published:** 2013-08-21

**Authors:** Amber L Pearson, Philippe Apparicio, Mylène Riva

**Affiliations:** 1 Department of Public Health, University of Otago, PO Box 7343, Mein Street, Newtown, Wellington 6242, New Zealand; 2 Centre Urbanisation Culture Société, Institut National de la Recherche, 385, rue Sherbrooke Est, Montréal, Québec H2X 1E3, Canada; 3 Centre de Recherche du CHUQ, Université Laval, 2875 Boulevard Laurier, Édifice Delta 2, bureau 600, Québec G1V 2M2, Canada

**Keywords:** Deprivation, Trends, Accumulation

## Abstract

**Background:**

Area-level socioeconomic deprivation has been shown to exert an independent effect on both individual and population health outcomes and health-related behaviours. Evidence also suggests that health and economic inequalities in many countries are increasing in some areas but may be on the decline in others. While area-level deprivation at a single point in time is known to influence health, the literature relating to longitudinal deprivation of communities and associated health impacts is sparse. This research makes a methodological contribution to this literature.

**Methods:**

Using a Latent Class Growth Model, we identified 12 deprivation trends (1991–2006) for small areas (n = 1621) in New Zealand. We then fitted regression models to assess the effects of trends of relative deprivation on a) all-cause mortality, and b) cardiovascular mortality (2005–2007) by census area unit. For comparison, we also fitted regression models to assess the effect of deprivation deciles (in 2006) on outcomes a) and b).

**Results:**

Using trends, we found a positive association between deprivation and mortality, except for two trends for both all-cause and CVD-related mortality. When comparing trends and deciles of deprivation, we observed similar patterns. However, we found that AIC values were slightly lower for the model including deciles, indicating better model fit.

**Conclusion:**

While we found that current deprivation was a slightly better predictor of mortality, the approach used here offers a potentially useful alternative. Future deprivation research must consider the possible loss of information about health benefits of living in areas where relative deprivation has improved in cross-sectional analyses.

## Background

Health inequalities, steeped in underlying social and economic inequalities, are on the rise in some countries while on the decline in others [1-3]. Within the vast evidence supporting the relationship between deprivation and health, a subset involves area-level socioeconomic deprivation. An important research focus involves understanding the complex and interactive feedback between composition and context in deprived neighbourhoods [4-7]. Classification of an area as ‘deprived’ can be the result of high concentrations of low-income residents. Equally, a dearth of employment opportunities in an area or hubs of affordable or state-owned housing can lead to the characterisation of some neighbourhoods as deprived. In this way, changes in neighbourhood deprivation may be the result of changes in neighbourhood composition or context. Indeed these processes are iterative and non-mutually exclusive. Due to the extensive evidence on the links between area deprivation and health, changes in deprivation and associated health consequences may have significant relevance for policy and resource allocation. Understanding the drivers of those changes (whether context or composition or both) may also be important to consider.

Studies tend to evaluate associations between current or past area-level socioeconomic conditions and health, but could benefit from considering multiple time points. While examination of health over time is well-established, few studies have examined neighbourhood deprivation in this way [8]. Since most area-level studies evaluate socioeconomic conditions at a single time point, or in two, to understand health differences between periods [9], little is known about the health impacts of the changes in neighbourhood deprivation over multiple time periods or trends of deprivation in places.

Ecological research can aid in understanding how longer term neighbourhood conditions or population compositions of neighbourhoods may contribute to population health outcomes in those areas. For example, Riva and Curtis found higher risk of premature mortality and limiting long-term illness in areas of England with persistently low or declining employment rates, compared to other areas [10]. They concluded that trends in area-level employment rates were slightly better predictors compared to analyses measuring employment rates a single time point. While employment rates are an important component of area deprivation, a composite measure may encompass other socioeconomic factors important to health. As such, area deprivation at multiple time points could provide ‘trends’ of deprivation, to be examined in light of current neighbourhood health as a way of understanding potential accumulation of disadvantage in places. Trends of deprivation may be stable or may indicate increases/decreases in relative position over time. Health may be influenced by the change in deprivation rank itself or the directional movement, regardless of rank. Understanding changes in the socioeconomic composition of areas, and whether they have either improved or worsened, may provide useful insight for health promotion. The literature relating to longitudinal deprivation of communities (rather than individuals) and associated health impacts is sparse. As such, this research makes a methodological contribution to this literature.

New Zealand offers a useful venue for examining changes in deprivation over time, as income inequalities have been higher in the last two decades than previously [11]. However, uniquely, deprived neighbourhoods in New Zealand tend to have equitable access to many amenities important to health [12] and thus, increases in deprivation may not have such dire consequences on health as in other places.

The trends approach has the advantage of permitting the examination of both the change in relative conditions over time and the directional trend itself. The objectives of this study were threefold. First, we categorised the trends of relative deprivation from 1991 to 2006 at the census area unit level (hereafter CAU), which are useful approximations of a neighbourhood, particularly in urban areas. Second, we examined the associations between trends and all-cause and cardiovascular-related (CVD) mortality (smoothed 2005–2007) for each CAU. Finally, we compared results between trends and 2006 deciles of deprivation (standard analysis). In a similar approach to Riva and Curtis [10], we evaluated the numbers of significant associations (for categorical deciles and trends), log likelihoods and AIC values to compare regression models using trends versus deciles of deprivation.

## Methods

### Geographic level of analysis

This national study involved analysis at the CAU level over four census years: 1991, 1996, 2001 and 2006. The CAUs represent a relatively small geographic unit, approximating a neighbourhood (Statistics New Zealand, [13]). Area and population sizes vary among the CAUs, especially between rural and urban areas (mean population = 2267; median = 2124; sd = 1581; mean size = 147 km^2^; median size = 3 km^2^; sd = 534 km^2^).

Moreover, geographic boundaries of several CAUs changed between 1991 and 2006; the number of CAUs increased from 1637 in 1991 to 1784 in 2006. While most boundaries remained unchanged, some CAUs were split into two over time (due to population increases). In order to obtain a repeated cross-sectional dataset with the same spatial units throughout the period, we harmonised geographic boundaries by aggregating contiguous CAUs which were split at some point after 1991. Next, we summed the count variables and generated population-weighted averages of deprivation scores. In this way, we obtained a total of 1621 CAUs with identical boundaries across the fifteen year period.

### Health data and potential confounder data

We compiled all-cause and CVD mortality from the Ministry of Health, measured as counts by five age groups (0–4, 5–24, 25–44, 45–64, and over 65 years) for each CAU. We smoothed the data by averaging death counts for each age group for periods between January 2005 and December 2007 (inclusive) to arrive at one value per CAU for the period. We also compiled area-level percentage smoker from the 2006 census, as we wanted to examine the role of this variable as a potential confounder of the association between area deprivation and mortality.

### Creation of the trends of deprivation – latent class growth modeling

A time series of area-level deprivation (NZDep) was compiled, which was originally generated from 1991, 1996, 2001 and 2006 census data. The variables included in NZDep have changed minimally over time. For example, NZDep1991 included 10 variables, which dropped to nine variables in NZDep1996, eight of which were common in both. Also, a few questions and classifications on census have changed slightly (e.g., the crowding variable changed in 2001). For a detailed discussion of NZDep and comparisons over time, see [14]. The implications of these changes to NZDep for this research were considered minimal because: (i) NZDep is designed to be an indicator of relative neighbourhood socioeconomic deprivation and therefore the small changes in the measure should still be capturing relative deprivation at each time point; and (ii) NZDep has been validated and used in health research at each time point in our study period. Variables comprising NZDep include employment status, income, single-parent households, education levels, crowded households, home, telephone and car ownership and uptake of government assistance programs [15]. We then ranked raw deprivation scores for each CAU for each census (where higher scores indicate higher deprivation) to minimise issues related to comparison over time, as advised by the creators of NZDep [14]. NZDep is created using small areas each containing roughly 100 people, which are conglomerations of meshblocks (the smallest aggregate unit in New Zealand). All meshblocks within each small area are then assigned the same NZDep score. With each census, different small areas were generated to create NZDep, as there may be changes in the size of the population and the occupiers of homes. As such, comparisons of deprivation by meshblocks would be inappropriate. Also, comparison of raw scores is not appropriate as maximum values also change. Instead, we were interested in large changes in rank over time. We produced a dataset of ranked deprivation at four time points for 1621 CAUs.

To identify the trends of deprivation, we applied a Latent Class Growth Model (LCGM). LCGM is a semi-parametric statistical technique designed for classifying longitudinal data [16-18]. For example, it has been used to group individuals with similar trends of change in health-related behaviours [19] and similar trajectories of social mobility [20]. While LCGM has mainly been applied to individual data, particularly in psychology and epidemiology, this method has recently been applied to spatial data [10,21]. Due to the difficulty of building a longitudinal, spatial dataset, few studies have applied this method to area units [10]. We used LatentGOLD software [22] to classify CAU in four to 20 clusters, as we had no *a priori* assumption about the optimal number of trends. The use of the LCGM to repeated cross-sectional, area rank deprivation-type data is relatively novel, and the pattern of findings for flat versus upward or downward trajectories may be quite novel, and therefore of interest. Compared to most applications of LCGM-style analysis quite a large number of trends were found.

### Statistical analyses

Associations between trends of deprivation and mortality was assessed using negative binomial regression model because of evidence of over dispersion of the mortality counts. These models are conceptually similar to Poisson regression models, but account for over-dispersion of the dependent variable (counts) in the calculation of standard errors for model coefficients. We included counts of smoothed, all-cause or CVD-related deaths by age group as the dependent variable, the age-specific population count as an offset (to allow modeling of rates rather than counts), and categorical deprivation trends as the independent variable (which has 11 parameters representing the non-reference levels of this factor). Incidence rate ratios (IRRs) for the deprivation trends were derived from these negative binomial models by exponentiating the model coefficients.

Traditional analyses use a snapshot of area-level deprivation to predict a wide array of health outcomes and behaviours [23]. So, for comparison, we also fitted two models which included similar variables, except categorical NZDep2006 deciles (nine parameters representing the non-reference levels of this factor) were used as the independent variable in place of the deprivation trends. To compare model fit, we examined AIC values.

We also fitted the models adjusted for area-level percentage regular smokers. This variable was however not significantly associated with the outcome variables (at the conventional p value <0.05), so it was not included in the final model. Analyses were conducted in Stata v.12 [24].

## Results

For the 2005–2007 time period, there were a total of 49,543 deaths, of which 19,790 were CVD-related (40%). There was an average of 6.1 deaths per CAU and 2.4 were CVD-related. Forty-three percent of all-cause and 46% of CVD-related deaths occurred in the age group 65 years and over.

**Figure 1:**
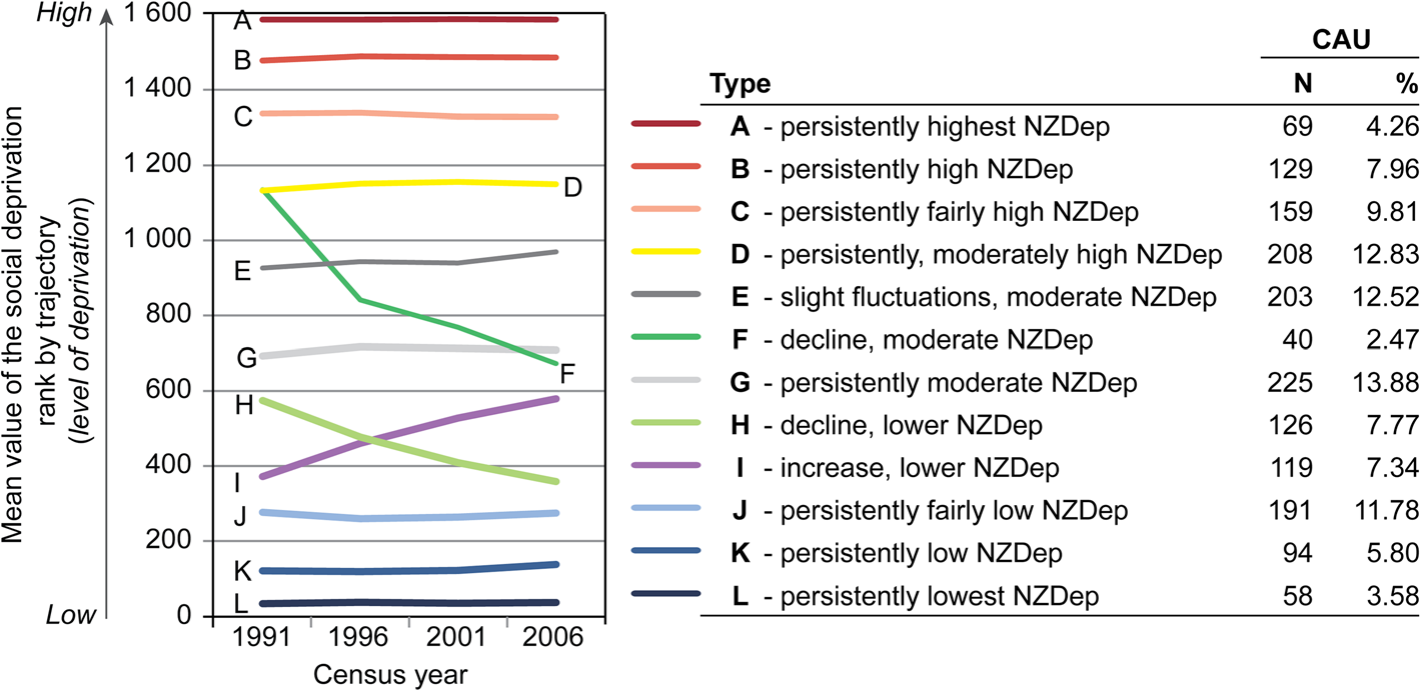
Trends of relative social deprivation between 1991 and 2006 obtained by the LCGM method.

**Figure 2:**
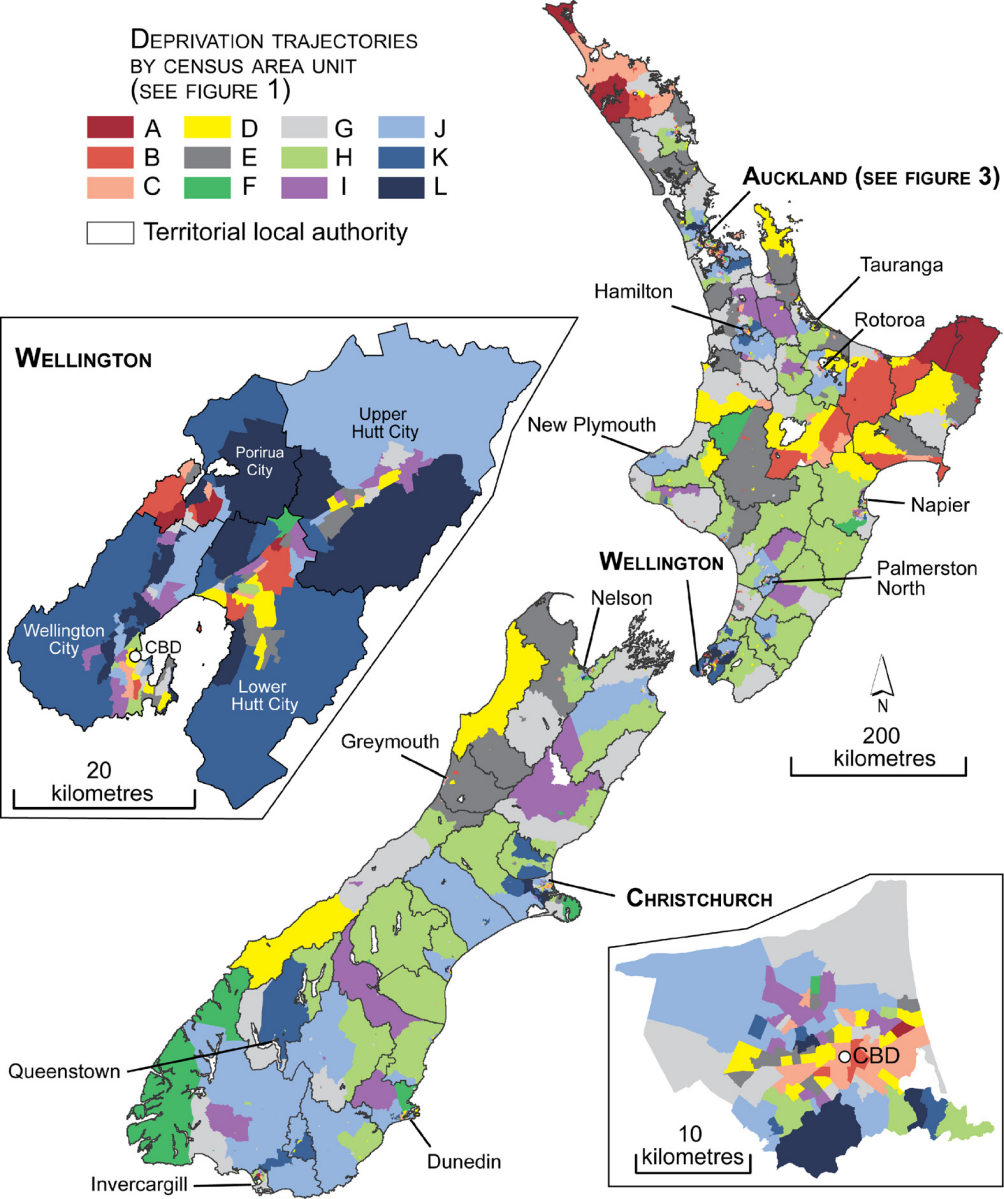
Map of trends of deprivation (1991 and 2006) obtained by the LCGM method.

**Figure 3:**
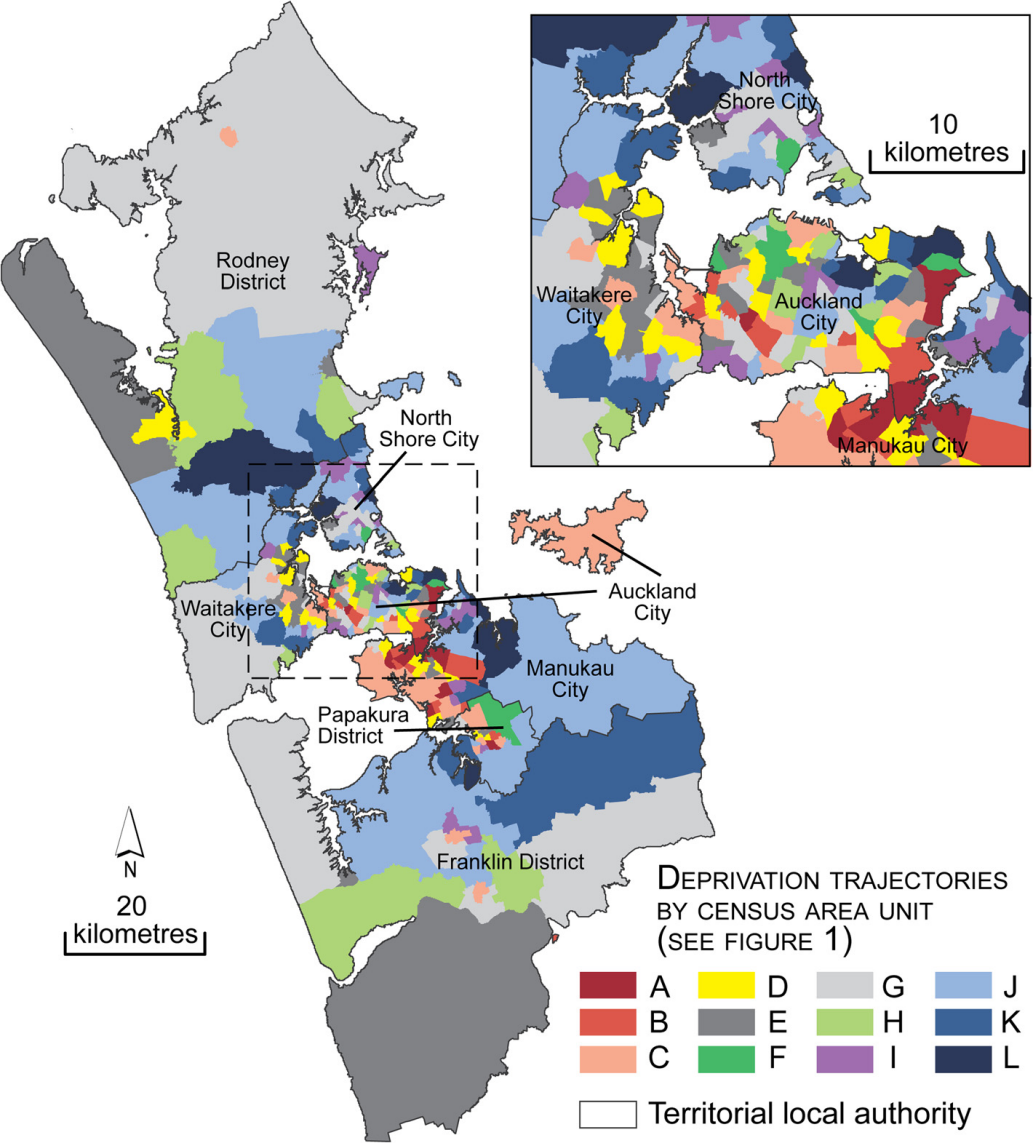
Map of trends of deprivation (1991 and 2006), Auckland region.

When creating the trends, the fit statistics (the lowest Bayesian Information Criterion value) indicated that the 1621 CAUs were optimally classified into 12 trends (i.e. groups of CAU having followed similar trends in deprivation between 1991 and 2006). However, some of these groups comprise less than 5% of CAUs and therefore could be considered ‘sparse’. Yet we chose to keep all twelve as they were characterized by ascending and descending trends of deprivation. When limiting the groups to ten or fewer, most trends were stable, since areas with dramatic changes in deprivation were few, as could be expected. The twelve deprivation trends over the study period were plotted using mean values of the deprivation ranks for each class and mapped (see Figures 1, 2 and 3). Most trends were relatively stable over the 15 year period, representing persistently low (J, K and L), moderate (G) or persistently high (A, B, C and D) deprivation rankings. However, one trend represented slight fluctuations over time (E). Two trends represented a decline in deprivation rankings (F and H); and one trend (I) represented a large increase in deprivation ranking. Geographically, there was no clear patterning of trends. The areas with decreasing deprivation trends (F and H) were found predominantly in rural areas on both islands and minimally within Auckland, Wellington and Christchurch. With the exception of Christchurch City, Invercargill and Dunedin, all of the persistently highly deprived areas were found on the north island, both in rural and urban areas. Areas of dramatically declining deprivation (trend F) were located mostly in central Auckland in Freemans Bay and Westmere and Tamaki, all of which have experienced gentrification. In rural areas, trend F was in peri-urban areas north of Dunedin which have been affected by the establishment of Macraes gold mine in 1990 and its continued expansion, the increasingly populated areas of Akaroa and the Napier wine growing region. Finally, the CAUs characterized by increasing deprivation (increasing poverty; trend I) were located on both islands and mainly in the suburban areas of the three main cities.

Results of the regression analyses are presented in Table 1. When using categorical variables, the reference category would ideally have a reasonable number of observations to maximise comparability between categories; thus, we used trend G which had relatively stable, moderate deprivation over time and consisted of 225 CAUs. For the persistently higher trends (A-D), incidence rate ratios (IRRs) of both all-cause and CVD mortality indicated significantly increased incidence, compared to the reference moderate trend (G). The other moderate trend (E) also had a significant and higher IRR than the reference trend for both outcomes. The findings for the declining trends (F, H) were not consistent. For trend F, we did not detect any significant associations compared to mortality rate ratios in areas characterised by stable and average levels of deprivation over the period (G). However, for trend H, we found significantly lower IRRs for both all-cause and CVD mortality. For the inclining trend (I) the IRRs were not significant. For both trends F and I, at the start of the period, the deprivation was higher for F and much lower for I, whereas their ranks were very close to G (reference group) at the end of the period. Thus, these associations were not significant. The opposite was observed for descending trend H, which had a similar rank value to G in 1991 and a lower one in 2006. Thus, we detected 13% lower mortality for trend H (IRR = 0.87), compared to the reference group. For the persistently low trends (J-K), we observed decreased IRRs for both outcomes and these were significant for trends J and L.

**Table 1:** Results of negative−binomial regression models

		**All−cause mortality**			**CVD−related mortality**	
		**(age−adjusted model)**			**(age−adjusted model)**	
	**IRR**	**(95% CI)**	**Z−statistic**	**IRR**	**(95% CI)**	**Z−statistic**
**Trends of relative deprivation**						
L − persistently lowest NZDep	0.75	(0.67−0.84)***	−5.19	0.80	(0.68−0.94)**	−2.71
K − persistently low NZDep	0.92	(0.84−1.00)	−1.85	0.99	(0.87−1.12)	−0.15
J − persistently fairly low NZDep	0.81	(0.75−0.87)***	−5.87	0.82	(0.74−0.92)***	−3.57
I − increase, lower NZDep	0.96	(0.89−1.04)	−0.98	1.05	(0.94−1.17)	0.82
H − decline, lower NZDep	0.87	(0.80−0.95)***	−3.19	0.86	(0.75−0.98)**	−2.29
G − persistently moderate NZDep	*Ref*			*Ref*		
F − decline, moderate NZDep	1.09	(0.95−1.24)	1.18	1.13	(0.92−1.39)	1.16
E − very slight fluctuations, moderate NZDep	1.17	(1.10−1.25)***	4.69	1.18	(1.07−1.30)***	3.28
D − persistently , moderately high NZDep	1.26	(1.18−1.34)***	6.93	1.24	(1.12−1.36)***	4.31
C − persistently fairly high NZDep	1.34	(1.25−1.44)***	8.44	1.30	(1.17−1.44)***	5.00
B− persistently high NZDep	1.51	(1.40−1.63)***	10.75	1.43	(1.27−1.61)***	6.06
A − persistently highest NZDep	1.68	(1.54−1.85)***	11.09	1.68	(1.46−1.94)***	7.08
Log likelihood		−9867.45			−5150.66	
AIC		19768.9			10335.32	
LR Chi^2^		1315.15			433.78	
Prob > chi^2^		<0.000			<0.000	
**Deciles of deprivation index in 2006**						
1. Lowest deprivation	0.78	(0.72−0.84)***	−6.14	0.79	(0.70−0.89)***	−3.79
2	0.89	(0.82−0.96)**	−3.08	0.91	(0.81−1.02)	−1.68
3	0.85	(0.78−0.92)***	−4.05	0.85	(0.76−0.96)**	−2.59
4	0.94	(0.87−1.01)	−1.67	0.96	(0.86−1.08)	−0.63
5	*Ref*			*Ref*		
6	1.11	(1.03−1.19)**	2.61	1.08	(0.97−1.21)	1.38
7	1.21	(1.12−1.30)***	4.99	1.16	(1.04−1.29)**	2.60
8	1.31	(1.22−1.41)***	7.45	1.29	(1.16−1.43)***	4.68
9	1.37	(1.27−1.47)***	8.36	1.31	(1.17−1.46)***	4.76
10. Highest deprivation	1.59	(1.47−1.71)***	12.18	1.53	(1.36−1.71)***	7.24
Log likelihood		−9861.44			−5147.90	
AIC		19752.89			10325.79	
LR Chi^2^		1312.61			438.01	
Prob > chi^2^		<0.000			<0.000	

* p < 0.05; ** p < 0.01; *** p < 0.001.

NOTE: Z−statistic = coefficient/standard error. The value follows a standard normal distribution which is used to test against a two−sided alternative hypothesis that the coefficient is not equal to zero.

When comparing these results with more standard analyses, (i.e. using NZDep deciles, with decile 5 as the reference category), we observed similar results, with significantly lower all-cause mortality, and mortality from cardiovascular diseases for areas in lower deciles of deprivation (deciles 1–3). We also observed slightly better model fit in the model including deciles of deprivation, as evidenced in marginally lower AIC values (<1% difference between the models).

## Discussion

In this research, we created categorical trends in area-level deprivation from 1991 to 2006 for all of New Zealand. We then tested the relationship between these categorical trends and all-cause and CVD-related mortality rates for those areas. We found that most trends were significantly associated with the mortality outcomes, with the persistently high and persistently low trends indicating increased and decreased incidences, respectively. We also found that the only inclining trend was not significantly associated with the mortality outcomes. We found that one of the declining trends (H) was associated with significantly lower mortality than the reference moderate and stable trend, while one (F) was not. Areas in trend H tend to be rural areas, often in parts of New Zealand which have experienced increases in dairy and wine production. While the influences are likely complex, this may be an example of the middle class rising in these areas. Although the most marked decline in deprivation occurred in trend F, this was not associated with either measure of mortality. We postulate that these areas experienced financial improvement in rural and gentrification in urban settings, displacing poorer households to other areas. In addition to the decline in deprivation in trend F, these areas also exhibited lower mortality. At the beginning of the period (1991), the deprivation level in declining trend F was similar to that in trend D. At the end of the period, the deprivation level of trend F was similar to that of the reference group, making it difficult to detect a significant association. The all-cause mortality incident rate ratio (IRR) of trend D was 1.26; whereas the IRR for declining trend F was 1.09. If the lower mortality was not due to declining deprivation, we would expect similar IRRs in trends F and D.

In comparison with more ‘typical’ analyses using cross-sectional deciles of deprivation, we found that our trend measures of deprivation did not fit the model as well, as indicated by slightly higher AIC values. However, these differences between the two models were marginal, suggesting that both approaches in measuring area deprivation in relation to area-level mortality may yield similar results. Our find that using current deprivation level slightly improved model fit may relate to the primarily stable levels of deprivation over the 15-year study period. These findings could also indicate that using longitudinal measures of mortality over time may be useful in future deprivation trend analyses. Since there are a number of factors aggregated in the deprivation index, one factor could improve while another one worsens over time, yet this could result in no net change in the deprivation index value. This information about material changes could be lost when using an index in relation to health measures. In contrast to our findings, research by Riva and Curtis found that trends in area-level employment rates were (slightly) better predictors of limited long-term illness and premature mortality, measured at the individual-level, than analyses measuring area-level employment rates at one point in time [10]. While our results did not show a major improvement in model fit of trends over deciles, we were able to observe a statistically significant, effect for the declining trend (H). Such information about health benefits of living in areas where relative deprivation has improved over time may be lost in cross-sectional analyses.

Several strengths and limitations are important to note. The primary strength of this research is its methodological contribution to the scant literature relating to longitudinal deprivation of communities and associated health impacts. In terms of potential criticism of the use of LCGM methods for inferential analysis, the defensibility of using identified trends or clusters as predictors in regression analyses has been established in other research (e.g., [25]). In terms of limitations specific to this study, this was an ecological study and, therefore, limitations include the inability to draw conclusions about individuals. In addition, the study does not take into account on migration of people or the length of residence in a particular place. Second, large changes in neighbourhood deprivation are rare events. Therefore, the latent class growth modelling method may not be suitable for identifying classes of rare events. To be detected, the technique may require large numbers. Stronger trends would also help elucidate relationships. Similarly, the length of the study period (15 years) may not be long enough for dramatic changes in deprivation levels to occur (e.g. processes of gentrification). The start of our study period was selected as it marks the first year that area-level deprivation (NZDep) was measured. Data for more time points, such as annually collected data, or for a longer time period would improve the current study. This approach may be most useful in urban settings to identify the processes above, or the mobility of people (e.g., poor, urban migration). Last, changes in NZDep may be due to the selection of spatial units (the modifiable area unit problem [26]), or changes in the actual population composition characteristics [14]. Some caution must be used when interpreting comparisons over time. However, we have attempted to minimise these sources of error by using the census area unit level, using ranked deprivation as the measure of comparison and in examining large changes in deprivation rank only. More sophisticated work could use individual-level health outcomes and deprivation trends of both individuals and areas over the life course to aid in examining the influence of mobility on these relationships and to get closer to understanding the dynamics of disadvantage accumulation in places and in individuals. This work suggests that further work is needed to understand the potential health benefits of living in areas where relative deprivation has improved over time.

## Conclusion

We found that most categorical deprivation trends, created for this research, were significantly associated with the mortality outcomes. Most associations were as expected, with the persistently high and persistently low trends indicating increased and decreased incidences, respectively. Several trends did not yield significant associations. However, we did find that one of the declining trends was associated with significantly lower mortality than the reference trend, which had a lower deprivation level over the study period. In comparing our categorical deprivation trend results with more ‘typical’, cross-sectional deprivation results, we found similar results, but better model fit when using cross-sectional deprivation declines. We conclude that categorical deprivation trends and cross-sectional deciles yielded similar results. Future research must consider that, in some cases, information about health benefits of living in areas which are improving in socioeconomic status may be lost when using cross-sectional deprivation data.

## Competing interests

The authors do not have any competing interests.

## Authors’ contributions

AP and PA conceptualised the study and performed analyses. AP drafted the manuscript. MR advised on analytical strategies. All authors edited the manuscript. All authors read and approved the final manuscript.
